# Are off-target effects of imatinib the key to improving beta-cell function in diabetes?

**DOI:** 10.48101/ujms.v127.8841

**Published:** 2022-09-14

**Authors:** Nils Welsh

**Affiliations:** Department of Medical Cell Biology, Uppsala University, Uppsala, Sweden

**Keywords:** Pancreatic beta-cells, type 1 diabetes, type 2 diabetes, imatinib, tyrosine kinase inhibitor, mitochondria

## Abstract

The small tyrosine kinase (TK) inhibitor imatinib mesylate (Gleevec, STI571) protects against both type 1 and type 2 diabetes, but as it inhibits many TKs and other proteins, it is not clear by which mechanisms it acts. This present review will focus on the possibility that imatinib acts, at least in part, by improving beta-cell function and survival via off-target effects on beta-cell signaling/metabolic flow events. Particular attention will be given to the possibility that imatinib and other TK inhibitors function as inhibitors of mitochondrial respiration. A better understanding of how imatinib counteracts diabetes will possibly help to clarify the pathogenic role of beta-cell signaling events and mitochondrial function, and hopefully leading to improved treatment of the disease.

## Introduction

For some 20 years now, the small tyrosine kinase inhibitor (TKI) imatinib mesylate (Gleevec, STI571) has been successfully used for the treatment of chronic myeloid leukemia and other malignancies. Imatinib targets certain oncogenes, such as Bcr-Abl, leading to apoptosis and decreased proliferation of malignant cells. In this context, it exerts its effects by binding to the Abl tyrosine kinase (TK) when it is in its closed and inactive conformation, which results in occlusion of the substrate site, a distorted Adenosine triphosphate (ATP)-binding site, and therefore, a dramatically reduced catalytic activity (1). Somewhat surprisingly, imatinib also protects against diabetes in animal models (2–10), and it has now been reported that imatinib mitigates both type 1 and type 2 diabetes in humans (11–13). As imatinib inhibits many cellular TK that have not been mutated into oncogenes, for example, c-Abl, Arg, PDGFR, c-Kit, DDR1, Flt-3, c-Src, and Lck, a multitude of imatinib-induced effects in non-transformed cells has been observed (14–18). In addition, imatinib is known to bind and inhibit also non-TK proteins, for example, the quinone oxidoreductase-2 enzyme, the ATP-sensitive K^+^ channel, and the V-ATPase (14, 19, 20). This occurs most probably by binding to ATP-pockets, in which the purine/pyrimidine moiety of imatinib fits. As such pockets are present in many proteins, the complexity of imatinib-induced effects increases considerably. Consequently, specific pivotal mechanisms by which imatinib ameliorates diabetes are not easily identified, and it is possible that many modest effects in concert result in the observed antidiabetic action. Indeed, a multitude of processes, such as peripheral insulin sensitivity, autoimmunity, inflammation, autophagy, fibrosis/amyloidosis, and arteriosclerosis (21–27), have been suggested to be modulated by imatinib, thereby providing a number of possible causes that either individually or synergistically promote improved metabolic control in diabetes. However, among the many pleiotropic actions of imatinib may of particular interest be imatinib’s role as a beta-cell protective drug, promoted by a direct effect on beta-cell signaling, and not via peripheral or indirect effects, a notion supported by findings both *in vitro* (2, 3, 28–33) and *in vivo* (5, 6, 13, 32). As beta-cells have a decisive role in glucose homeostasis, and loss of beta-cell function and survival is a crucial step in the pathogenesis of both type 1 and type 2 diabetes, it is tempting to speculate that this beta-cell protection effect could be an explanation of particular importance to the antidiabetes actions of imatinib. Therefore, this short review will focus on possible mechanisms, both TK-dependent and TK-independent, by which imatinib and other TKIs modulate signaling events and metabolic flow in insulin producing cells, and how this improves beta-cell function and survival.

## Imatinib-targeted TKs and their down-stream effectors in beta-cell survival

At first glance, it appears counterintuitive that a drug designed to promote apoptosis (of cancer cells) also promotes cell survival and improved function (of beta-cells), but it is important to note that non-malignant cells do not express imatinib-sensitive oncogene proteins and are therefore not highly prone to apoptosis when exposed to the drug. Instead, imatinib inhibits in primary cells a multitude of non-oncogenic TKs, among which c-Abl has received particular attention. This non-receptor and multifunctional TK (34) is inhibited by imatinib with an inhibitor concentration 50 (IC_50_) of 100–300 nanomolar, and in other cells than beta-cells, it is well known that c-Abl activation in response to different types of stress promotes apoptosis, an event which can be inhibited by treatment with imatinib (35–37). C-Abl-induced apoptosis seems to be achieved via interactions with, for example, ATM (38), TIP1 (39), Parkin (40), stress-activated protein kinases (41), p53 (42), p73 (43), and NF-kappaB (44). These proteins play important roles in tumor suppression (ATM, p53, p73), microtubule cytoskeletal organization (TIP1), protein ubiquitination (Parkin), and inflammation (NF-kappaB). It was also observed that c-Abl is partially localized to the endoplasmic reticulum (ER), and that ER stress promotes c-Abl activation and translocation to mitochondria where apoptotic signals leading to caspase 3 activation are initiated (45).

Much less is known about the role of c-Abl in insulin-producing beta-cells, but the c-Abl-activation seems to promote apoptosis/dysfunction in this cell type as siRNA-mediated c-Abl knockdown has been reported to reduce cell death rates of mouse islet cells (3) and the mouse insulin producing cell line βTC-6 (46) or improve beta-cell function of the mouse insulin producing cell line NIT-1 (29) and the rat insulin producing cell line INS-1 as well as the human insulin producing cell line EndoC-βH1 (30). Some beta-cell c-Abl down-steam targets, direct or indirect, have been inferred via siRNA and co-immunoprecipitation experiments, namely, the phosphatidylinositol-3, 4, 5-triphosphate (PIP_3_) phosphatase Inositol Polyphosphate Phosphatase Like 1 (INPPL1) and the phosphatidylinositol-3-kinase (PI3K) pathway, using EndoC-βH1 cells and the mouse insulin producing cell line MIN6 (28), the transcription factor Nkx2.2 (29), PDGFR/LRP1-induced ERK phosphorylation (30), the estrogen-related nuclear receptor gamma (ERRgamma), using INS-1 cells (47), and IRE-1alpha, one of the three arms of the unfolded protein response (UPR), using INS-1 cells and mouse and human islet cells (32). In the first case, we observed that c-Abl co-immunoprecipitated with INPPL1, and that the c-Abl/INPPL1 interaction was associated with decreased PI3K/PIP_3_ signaling (28). PIP3-signaling is known to reduce cellular apoptosis. Consequently, imatinib inhibited the c-Abl-induced suppression of the PI3K pathway, and the resulting imatinib-induced increase in Akt phosphorylation fits well with improved beta-cell survival, for example, via increased phospho-BAD or beta-catenin signaling (28). Second, c-Abl downregulation resulted also in increased expression of Nkx2.2, a positive regulator of insulin gene expression, which was associated with higher levels of the glucose transporter GLUT-2 (29). These findings support a role of imatinib as a stimulator of beta-cell function, in addition to merely maintaining survival. The mechanisms by which c-Abl downregulation resulted in increased Nkx2.2 were, however, not further characterized. Third, c-Abl appears to reduce PDGFR/LRP1-induced ERK phosphorylation, which could explain the pronounced activation of ERK that occurs in response to imatinib at basal conditions (30). ERK is known to positively regulate both beta-cell mass and function (48), which concurs with the finding that an ERK inhibitor partially blocked imatinib-induced protection against beta-cell death (30). Fourth, using the c-Abl inhibitor GNF2 on INS1 cells, it was demonstrated that basal c-Abl activity may suppress the expression of the orphan nuclear receptor ERRgamma, and that this leads to reduced glutaminase 1 activity and a weaker glutathione-dependent defense against oxidative stress (48). Fifth, activated c-Abl seems to directly bind to and activate IRE1alpha in beta-cells, leading to XBP1 splicing, ER stress, and beta-cell death in NOD mice (32). Therefore, imatinib, acting as a c-Abl inhibitor, protected against beta-cell ER stress and subsequent cell death by blocking the c-Abl/XBP1 interaction (32). On the other hand, it is noteworthy that imatinib also causes ER stress in cardiomyocytes via a c-Abl-independent pathway leading to cardiomyopathy, a known complication to imatinib therapy (49). Furthermore, it has recently been demonstrated that c-Abl promotes a type of IRE1alpha activation that mediates a DNA-damage response, rather than the canonical UPR that occurs in response to ER stress (50), indicating that the complex role of imatinib and c-Abl in ER stress may involve opposite actions. Nevertheless, it appears that c-Abl inhibition may positively modulate different beta-cell pro-survival and pro-function events. This is in line with the notion that c-Abl is a modulating, rather than a master switch type, TK that fine tunes the cellular outcome in response to stress (34). On the other hand, it cannot be excluded that hitherto unknown counter-regulatory events become activated by imatinib-induced TKI inhibition, and that such putative events may blunt or even nullify the pro-survival signals.

Other classical imatinib TK targets, such as c-Kit and the PDGF receptor, have received less attention than c-Abl when it comes to protection against beta-cell death and diabetes, but one report states that c-Kit is not necessary for imatinib-induced protection against diabetes in NOD mice (8).

## Imatinib-targeted non-TKs and their role in beta-cell survival

**Figure 1:**
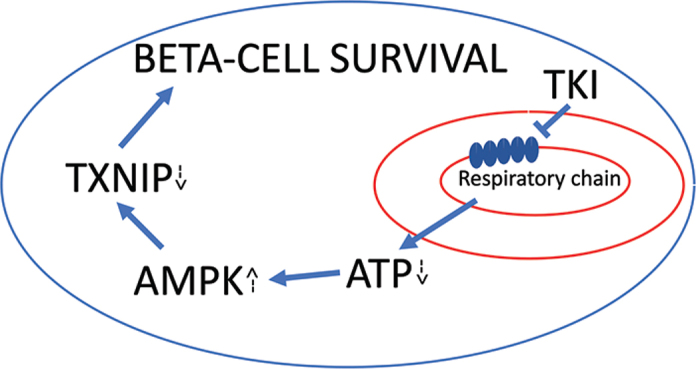
Possible off-target mechanism for tyrosine kinase inhibitors that promote improved beta-cell function and survival. See text for details.

As micromolar concentrations of imatinib are required for protection against beta-cell death and dysfunction *in vitro*, and as only high nanomolar concentrations are required for inhibition of the classical imatinib targets *in vitro*, it is possible that non-TK targets with IC_50_s in the micromolar range, i.e. off-targets, mediate a substantial part of the protective actions. Indeed, it has been observed that imatinib, in MIN6 cells, binds to and inhibits the ATP-sensitive K^+^-channel with an IC_50_ of 9.4 micromolar (19), probably via binding to the ATP-pocket. In the same study, it was also observed that the non-c-Abl inhibitor sunitinib promoted the same effect, suggesting that the effects were not mediated by c-Abl. Along the same line, we recently observed that both imatinib and sunitinib moderately inhibited complex I and II of the respiratory chain at micromolar concentrations when supplemented to isolated rat kidney mitochondria (33). The molecular details of imatinib binding to the respiratory complexes have not been characterized, but both complexes have NAD-binding pockets, and it is therefore possible that the purine moiety of many TKIs will fit as well. Indeed, using isolated rat liver mitochondria, it was observed that 18 out of 31 TKIs promoted toxic effects to the mitochondria, suggesting that the inhibition of respiration is a common trait of TKIs (51). In myocardial cells, it was observed *in vitro* that sunitinib induced mitochondrial inhibition at 5–10 micromolar concentrations, and that this resulted in oxidative stress and apoptosis (52), and *in vivo*, it has been observed that respiration was reduced, oxidative stress increased, and mitochondrial proliferation decreased (53). In HEK293 cells, sorafenib also has been demonstrated to inhibit respiration (54). Thus, it appears that multiple TKIs promote mitochondrial inhibition, and that this could be more or less a class effect (Figure 1).

Imatinib-induced inhibition of respiration in human beta-cells (EndoC-βH1 and human islets) resulted in lowered respiration rates, a decreased ATP/AMP ratio and AMP-activated protein kinase (AMPK) activation (33). Interestingly, the AMPK activator AICAR mimicked, whereas the AMPK inhibitor compound C counteracted the imatinib effect on survival (33), suggesting that imatinib acts on beta-cell survival, to some extent, via AMPK activation. AMPK is activated in response to lowered ATP/AMP ratios and rescues cells by increasing autophagy, catabolism, and energy conservation (55), and we have previously observed that AMPK activation in EndoC-βH1 cells promotes human beta-cell survival in response to pro-inflammatory cytokines, which are proapoptotic factors able to reduce beta-cell ATP-levels (56). It may be that AMPK mediates its protective actions, at least in part, via the downregulation of thioredoxin interacting protein (TXNIP) (33), a protein that is known to promote beta-cell dysfunction and death (57) (Figure 1).

Imatinib has recently been found to preserve beta-cell function in adults with recent-onset type 1 diabetes resulting in an initial lowering of the exogenous insulin dose as well as HbA_1c_ levels, an effect not associated with any clear alterations in immunity (13). The beneficial effects of imatinib were lost upon discontinuation of the treatment, suggesting that imatinib needs to be administered in the long term in order to protect beta-cells from dysfunction (13). In type 2 diabetes, imatinib also promotes a glucose-lowering effect (11, 12). Interestingly, not only imatinib but also other TKIs have been reported to promote antidiabetes effects. In different case reports, erlotinib, dasatinib, sunitinib, and sorafenib lowered the blood glucose in patients suffering from both cancer and diabetes (58–61). As these TKIs have different TK target profiles, it can be hypothesized that the anti-diabetic actions result from mitochondrial inhibition, or some other ‘off-target’ effect, rather than from inhibition of specific TKs with low IC_50_ values. Further support for this hypothesis may be provided by a recent report stating that dasatinib, a Bcr-Abl TKI, which is also considered to be a potent senolytic agent when combined with quercetin (62), promotes more pronounced anti-diabetic actions than imatinib in humans with type 2 diabetes (63). Interestingly, it has recently been shown that dasatinib in myotubes inhibits mitochondrial function more efficiently than imatinib at equimolar concentrations (64).

## Conclusions and future directions

Increasing evidence suggests that imatinib and other TKIs improve beta-cell function and survival, and although events via c-Abl signaling cannot be ruled out, it may be that this occurs mainly via ‘off-target’ effects that are TK-independent. Therefore, it is warranted to further characterize mitochondrial ‘off-target’ effects of TKIs, and to compare them with those of other mitochondria-targeting drugs, for example, metformin (65) or the mitochondrial K(ATP)-channel opener NNC 55-0321 (66). Finally, it is noteworthy that the moderately efficient antidiabetes agent imatinib protected against recent-onset type 1 diabetes equally well or even better than many immune-targeting therapies (13), which raises the interesting question whether the more potent mitochondrial inhibitor dasatinib would perform even better as a treatment for type 1 diabetes.
